# Development of Highly Sensitive Biosensors of RAF Dimerization in Cells

**DOI:** 10.1038/s41598-018-37213-2

**Published:** 2019-01-24

**Authors:** Kyoko Miyamoto, Masaaki Sawa

**Affiliations:** 1 CarnaBio USA, Inc., 329 Oyster Point Boulevard, Suite 300, South San Francisco, CA 94080 USA; 2 Carna Biosciences, Inc., 1-5-5 Minatojima-Minamimachi, BMA 3rd Floor, Chuo-ku, Kobe 650-0047 Japan

## Abstract

The BRAF inhibitors dabrafenib and vemurafenib induce remarkable clinical responses in patients with BRAF-mutated melanomas. However, adverse events, including the emergence of secondary tumors and drug resistance, have been reported. Studies have revealed that undesirable RAF dimerization induced by inhibitors promotes these adverse effects. Here, we developed highly sensitive biosensors of RAF dimerization in cells utilizing the split enhanced click beetle luciferase (Emerald Luc, ELuc) complementation technique. We demonstrated that our biosensor system works effectively for high-throughput screens in the microplate format. A comprehensive analysis of commercially available RAF inhibitors performed using this assay system revealed that the inhibitors exhibit various potencies in inducing the dimerization of RAF isoforms, and their dimerization potencies do not always correlate with the RAF enzyme inhibition. This sensitive assay system will become a powerful tool to discover next-generation BRAF inhibitors with safer profiles.

## Introduction

RAF protein kinases are key components of the RAS-RAF-MEK-ERK signalling cascade, which transmits signals from cell-surface receptors to promote cell proliferation and survival^1,2^. Among three isoforms of RAF [ARAF, BRAF, and CRAF (also known as RAF1)], BRAF has attracted the greatest attention as a therapeutic target, since BRAF mutations have been identified in 8% of human tumors, particularly in ~60% of melanomas^1,2^. The most common BRAF mutation is BRAF(V600E), which has been detected in greater than 90% of BRAF-mutant tumors^1,2^. The ATP-competitive RAF inhibitors vemurafenib and dabrafenib show remarkable clinical activities in patients with BRAF(V600E/K) melanoma and received US Food and Drug Administration (FDA) approval for the treatment of this disease^2^. However, continuous administration leads to drug resistance and tumor relapse^2^. Moreover, vemurafenib and dabrafenib often produce undesirable side-effects, such as cutaneous squamous cell carcinoma, presumably due to the paradoxical activation of ERK signalling in normal cells^1^. Thus, a high demand exists for the development of more effective and safer BRAF inhibitors, and several second-generation BRAF inhibitors are under preclinical and clinical development^2^.

RAF dimerization is required for its activation (the transactivation of its counterpart) in normal cells and in RAS mutant-driven tumors, and dimerization is promoted in a RAS-dependent manner^3,4^. In addition, RAF dimerization is involved in the mechanisms by which inhibitors induce the paradoxical activation of ERK signalling^5–7^. Namely, inhibitors promote RAF dimerization, resulting in the activation of the counterpart RAF and the downstream MEK-ERK signalling. Substantial paradoxical activation of this pathway has been observed in BRAF-wildtype cells where RAS is active, while minimal activation of this pathway is observed in cells expressing the BRAF(V600E) mutant which functions as a monomer and does not require active RAS^3^. Furthermore, the recent study has reported that RAF dimerization promotes the development of drug resistance^8^. Thus, preventing RAF dimerization is an effective strategy for the development of inhibitors with improved safety and durable efficacy and could provide clinical benefits to patients with cancer driven by not only BRAF mutations but also RAS mutations on the BRAF-wildtype background.

Split luciferase complementation is a rapid and quantitative assay system for the detection of protein-protein interactions. Luciferase proteins that are split into amino- and carboxy-terminal halves reconstitute catalytically active luciferase when the fused proteins of interest interact and bring the two luciferase fragments in close proximity. Luciferase proteins from different species, such as firefly^9^, *Renilla*^10^, and the click beetle^11^, have been used for the split luciferase system. The recent study by Peng *et al*. used split firefly luciferase to detect RAF dimerization and demonstrated that a pan-RAF inhibitor LY3009120 promotes RAF dimerization with minimal paradoxical activation^12^. In contrast, in the present study, we utilized split Emerald Luc (ELuc), an enhanced luciferase from the click beetle (Brazilian *Pyrearinus termitilluminan*)^11,13^, and developed novel biosensors designed to monitor RAF dimerization in cells. Previous studies showed that ELuc emits significantly brighter luminescence than firefly luciferase in full-length^13^ and in complementation fragments^11^. Accordingly, we could develop highly sensitive probes to detect RAF dimers. We generated the comprehensive monitoring system to detect BRAF, BRAF(V600E), and CRAF hetero- and homodimers and examined the dimerization efficacies, in terms of potency and dimerization rate, of commercially available RAF inhibitors. The effects of those inhibitors on RAF enzyme activities were also examined to determine the relationship between dimerization and enzyme inhibition. Moreover, the effects of those inhibitors on downstream signalling in cancer cell lines harbouring BRAF, KRAS, or EGFR mutations were evaluated to assess the relationship between dimerization and paradoxical activation.

## Results

### Generation of RAF dimerization-sensing probes

**Figure 1:**
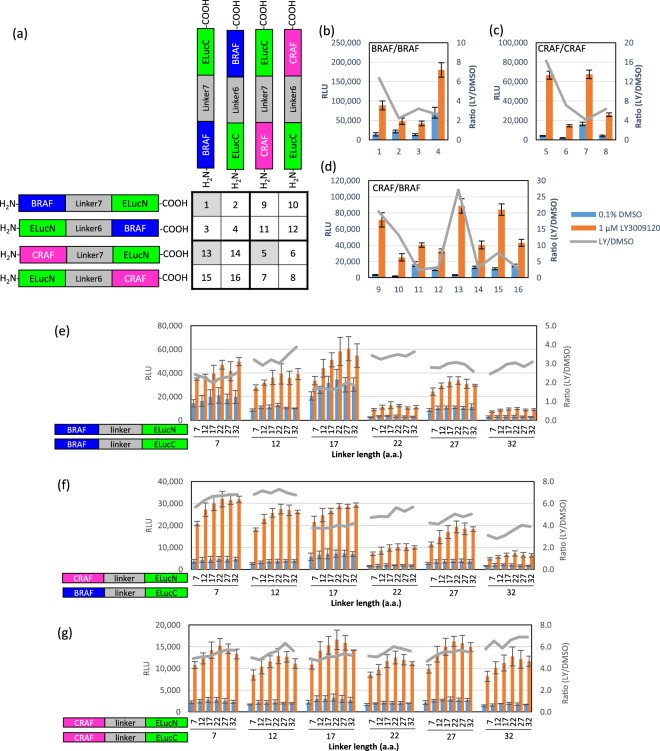
Development of split luciferase probes for detecting RAF dimerization. (**a**–**d**) Identification of the best probe pairs for detecting RAF dimerization using different orientations of RAF and ELuc fragments. 293T cells were transiently co-transfected with the indicated probe pairs (**a**), stimulated with 1 µM LY3009120 or the vehicle control (0.1% DMSO) for 2 hours, and luciferase activities were measured (**b**–**d**). (**e**–**g**) Optimization of the linker length. 293T cells were transiently co-transfected with the indicated probe pairs, stimulated with 1 µM LY3009120 or the vehicle control (0.1% DMSO) for 2 hours, and luciferase activities were measured. Results are presented as relative luminescence units (RLUs) (means ± SD, n = 3) and the ratio of RLUs measured in LY3009120-treated cells to DMSO-treated cells.

We selected the split ELuc complementation assay system to detect RAF dimerization in cells because ELuc yields a very bright luminescence signal with low background^11,13^. We first designed eight probe constructs, as illustrated in Fig. 1a, to examine the effects of the orientation of ELuc fragments on the RAF dimerization-induced ELuc reconstitution and activity. We constructed expression plasmids encoding the amino-terminal half of ELuc (1–415, ELucN) and the carboxy-terminal half of ELuc (394–542, ELucC) fused to the amino- or carboxy-terminus of BRAF or CRAF via Linker6 (6 amino acids) or Linker7 (7 amino acids) (Fig. 1a and Supplementary Table S1). Sixteen pairs of expression plasmids for the probes were transiently co-transfected into 293T cells in a pairwise manner, and on the next day, the cells were treated with 1 µM LY3009120 for 2 hours to induce RAF dimerization. As shown in Fig. 1b–d, all probe pairs gave the increased the luciferase activity upon LY3009120 stimulation. Among the pairs, the highest signal-to-background (LY/DMSO) ratios were yielded by the probe pairs in which both of the ELuc fragments were fused to the carboxy-terminus of RAF proteins (#1 for BRAF/BRAF, #5 for CRAF/CRAF, #9 and #13 for CRAF/BRAF dimerization). Thus, probe pairs #1, #5, and #13 were selected for the optimization of the linker lengths to maximize the signal-to-background (LY/DMSO) ratios.

We next designed GS linkers of various lengths^11^ that connected RAF proteins and ELuc fragments to optimize the linker length in the probes (Supplementary Table S2), since the linker length might substantially affect the proximity of two ELuc fragments for reconstitution and the accessibility of RAF proteins for dimerization. Twenty-four expression plasmids with various linker lengths were constructed and tested for luciferase activity in a pairwise manner. As shown in Fig. 1e–g, the linker length of BRAF-ELucC, but not BRAF-ELucN, CRAF-ELucN, or CRAF-ELucC, strongly affected the luciferase activity. For BRAF/BRAF and CRAF/BRAF dimerization, BRAF-(Linker12)-ELucC paired with BRAF-ELucN or CRAF-ELucN of any linker length yielded the maximum signal-to-background (LY/DMSO) ratio associated with the high luminescence signals. For CRAF/CRAF dimerization, the probes with any linker lengths yielded similarly high signal-to-background (LY/DMSO) ratios and luminescence signals. Therefore, we decided to use Linker12 for BRAF-ELucC and all other probes in the subsequent experiments to simplify our procedure.

To confirm that the observed signals were derived from RAF dimerization but not the self-interaction of ELuc fragments, we first paired each probe with unfused ELuc fragments. Without the RAF domain, the probes did not exhibit any increase in luciferase activity after the LY3009120 treatment; the LY/DMSO ratio was approximately 1 (Supplementary Fig. S1). We next tested dimerization-impaired mutants BRAF(R509H) and CRAF(R401H)^14^. As expected, the probes introduced with the BRAF(R509H) or CRAF(R401H) mutation exhibited 30–50% reductions in the LY/DMSO ratio compared to the wildtype probes (Supplementary Fig. S1). Based on the results from these two experiments, we concluded that the increased luciferase activities caused by the LY3009120 treatment were derived from specific RAF/RAF interactions. Interestingly, when the activating mutation BRAF(V600E) was introduced into the probe, the dimerization levels in both the basal and the LY3009120-stimulated states increased (Supplementary Fig. S1), indicating that BRAF(V600E) acquires an increased dimerization potency, despite its activity in the monomeric state^3^. Cell viability was not affected by the transient transfection of these probe pairs (Supplementary Fig. S2).

### RAF inhibitors induce RAF dimerization at various levels

It should be important to measure the RAF dimerization ability (potency, level, and velocity) of the compounds using a common assay platform to predict the adverse effects of RAF inhibitors elicited by RAF dimerization. Previous studies have shown that RAF dimerization is induced by not only first-generation inhibitors, such as dabrafenib and vemurafenib, but also “paradox-breaking” 2nd-generation inhibitors, such as LY3009120 and TAK-632, but not PLX7904^3,12,15–17^. However, complete, comprehensive data obtained using the same platform are not available. Therefore, we examined ten commercially available RAF inhibitors [dabrafenib, vemurafenib, sorafenib (also known as BAY43-9006)^18^, regorafenib^19^, LY3009120, SB-590885^20^, MLN2480 (also known as BIIB-024 or TAK-580)^21^, TAK-632, PLX4720^22^, and PLX7904] (the chemical structures are shown in Supplementary Fig. S3) to compare their abilities to induce RAF dimerization.

**Figure 2:**
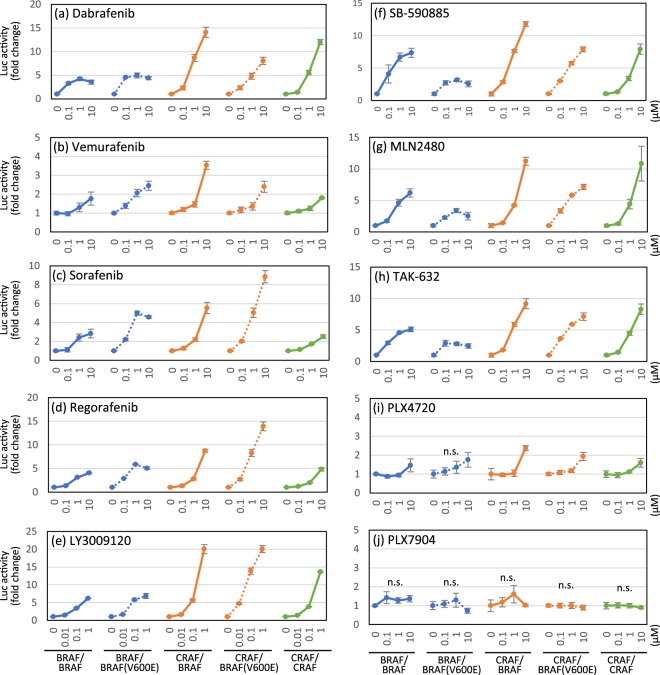
Quantitative analysis of RAF dimer induction by RAF inhibitors using split luciferase probes. 293T cells were transiently co-transfected with a pair of RAF split luciferase probe plasmids (Table 1), stimulated with the indicated concentrations of RAF inhibitors for 2 hours, and luciferase activities were measured. Results are presented as fold increases compared with the luciferase activity of vehicle (0.1% DMSO)-treated cells (means ± SD, n = 3). n.s., not significant; otherwise *P* < 0.05 (one-way ANOVA). Statistical data are provided in Supplementary Table S3.

We treated 293T cells that had been transiently co-transfected with the appropriate probe pair (Table 1) with various concentrations of RAF inhibitors for 2 hours, and then the luciferase activities were measured. The results are shown as fold increases in luciferase activity compared with the vehicle (0.1% DMSO)-treated cells to represent the inhibitor-induced dimerization level. Significant dimerization signals were induced by all RAF inhibitor treatments, except for PLX7904, and the dimerization levels range from a several- to a 20-fold increase, depending on the inhibitor and the probe pair (Fig. 2 and Supplementary Table S3). Among the compounds tested, LY3009120 showed the highest dimerization ability (Fig. 2e). Vemurafenib, the first FDA-approved BRAF inhibitor, displayed weak dimerization signals compared to other compounds (Fig. 2b). PLX4720 slightly but significantly promoted the dimerization of RAF probe pairs, except for BRAF/BRAF(V600E) (*P* = 0.056) (Fig. 2i), while PLX7904 induced no significant changes in the dimerization of any RAF probe pair at concentrations up to 10 µM (Fig. 2j). Among the RAF isoform pairs, CRAF-containing dimers [CRAF/BRAF, CRAF/BRAF(V600E), and CRAF/CRAF] were preferentially induced. In a comparison of the BRAF(V600E) mutant with the wildtype, dimerization levels of BRAF(V600E)-containing dimers were induced higher by sorafenib and regorafenib (Fig. 2c,d) and lower by SB-590885, MLN2480, and TAK-632 (Fig. 2f–h) than the levels of the wildtype dimers. These results reveal the different preferences of the RAF inhibitors for the induction of RAF homo-/heterodimers.

**Table 1:** Probe pairs used for the RAF split luciferase assay.

Probe Pair	Probe 1 (pcDNA3.1/myc-His)	Probe 2 (pcDNA4/V5-His)
BRAF/BRAF	BRAF-(Linker12)-ELucN	BRAF-(Linker12)-ELucC
BRAF/BRAF(V600E)	BRAF-(Linker12)-ELucN	BRAF(V600E)-(Linker12)-ELucC
CRAF/BRAF	CRAF-(Linker12)-ELucN	BRAF-(Linker12)-ELucC
CRAF/BRAF(V600E)	CRAF-(Linker12)-ELucN	BRAF(V600E)-(Linker12)-ELucC
CRAF/CRAF	CRAF-(Linker12)-ELucN	CRAF-(Linker12)-ELucC

To better understand the correlations between the dimerization potency of RAF inhibitors and their inhibitory potencies against RAF kinase activity, we measured the half-maximal inhibitory concentration (IC_50_) of each RAF inhibitor against RAFs and downstream MEK and ERK signalling at two different concentrations of ATP, near-Km (ATP_low_) and 1 mM (ATP_high_, a near-physiological concentration). At ATP_low_, all inhibitors showed submicromolar IC_50_ values against BRAF and CRAF (Supplementary Table S4). In contrast, at ATP_high_, those IC_50_ values were markedly increased, and the values of some inhibitors, such as vemurafenib, sorafenib, regorafenib, PLX4720, MLN2480, and TAK-632, exceeded 10 µM against BRAF (Supplementary Table S5). Nevertheless, those inhibitors significantly induced BRAF/BRAF dimerization at concentrations ≤10 µM (Fig. 2). Thus, the inhibitory potency does not always correlate with the RAF dimerization-promoting efficacy, suggesting the importance of performing a dimerization assay in addition to the enzymatic assay to profile the compounds.

### RAF inhibitors induce RAF dimerization with various temporal patterns

**Figure 3:**
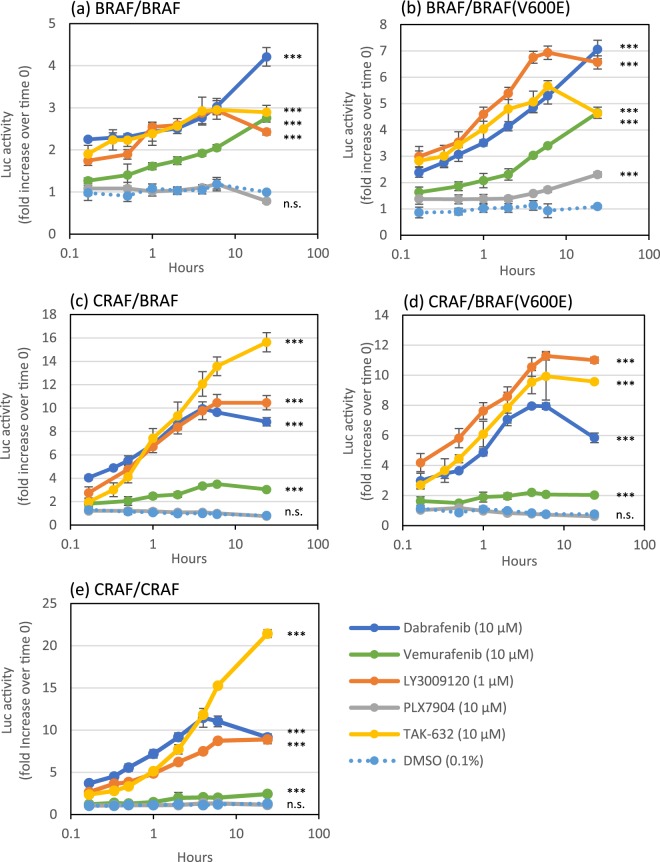
Temporal patterns of RAF dimerization promoted by RAF inhibitors. 293T cells were transiently co-transfected with a pair of RAF split luciferase probe plasmids (Table 1), stimulated with the indicated inhibitors for up to 24 hours, and luciferase activities were measured. The results are presented as fold increases compared with the luciferase activity measured at time 0 (means ± SD, n = 3). ***P < 0.001; n.s., not significant between inhibitor and DMSO treatments (two-way ANOVA). Statistical data are presented in Supplementary Table S6.

We next conducted time course experiments of inhibitor-induced RAF dimerization for selected RAF inhibitors (dabrafenib, vemurafenib, LY3009120, PLX7904, and TAK-632) to observe the temporal patterns of dimerization. Consistent with the results of the concentration-dependence study (Fig. 2), dabrafenib, vemurafenib, LY3009120, and TAK-632 induced the dimerization of all RAF isoform pairs to various degrees (Fig. 3 and Supplementary Table S6). In all RAF isoform pairs, dimerization induced by these four inhibitors was detected within 10 minutes (the minimum time point) and increased with different time courses, depending on the inhibitor and RAF isoform pair. Most dimerization reactions reached a peak or a plateau at 4–6 hours, while some continued to increase for up to 24 hours. For instance, dabrafenib and vemurafenib induced BRAF/BRAF and BRAF/BRAF(V600E) dimerization at increasingly higher levels for up to 24 hours (Fig. 3a,b), while the other three dimers reached a peak or a plateau at 4–6 hours (Fig. 3c–e). In contrast, TAK-632 induced CRAF/BRAF and CRFAF/CRAF dimerization at increasingly higher levels (Fig. 3c,e), while the other three dimers reached a peak or a plateau at 6 hours (Fig. 3a,b,d). LY3009120 exhibited quite similar changes over time (a peak or plateau at 6 hours) in all five dimerization (Fig. 3a–e). Finally, it is to be noted that the PLX7904 treatment slightly but significantly induced BRAF/BRAF(V600E) dimerization over time (Fig. 3b), which was not observed after a 2-hour treatment (Fig. 2j). Thus, the results indicate that the levels and the temporal patterns of dimerization vary, depending on the inhibitor and RAF isoform pair.

### Effects of RAF inhibitors on the MAPK pathway in cancer cell lines

**Figure 4:**
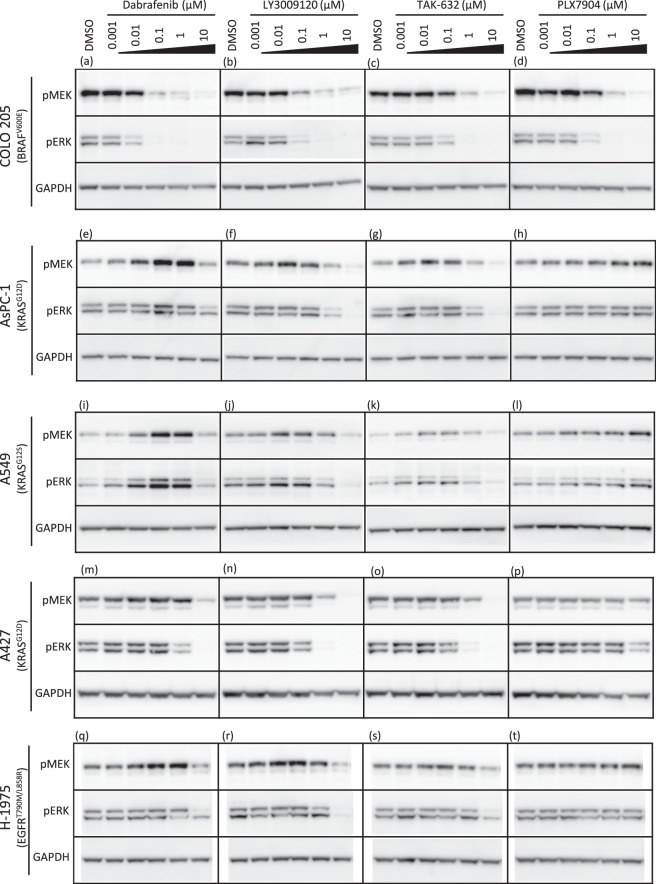
Effects of RAF inhibitors on the MAPK pathway in cancer cell lines. Phospho-MEK1/2, phospho-ERK1/2, and GAPDH levels in COLO 205 cells (**a**–**d**), AsPC-1 cells (**e**–**h**), A549 cells (**i**–**l**), A427 (**m**–**p**), and H-1975 cells (**q**–**t**) treated with dabrafenib, LY3009120, TAK-632, or PLX7904. Cells were treated with the indicated concentrations of inhibitors for 2 hours, and cell lysates were analysed by western blotting. Solid black lines represent the boundaries of images cropped from different blots. The membranes were cut out before blotting, and full-length blots are unavailable. Uncropped images are shown in Supplementary Fig. S5.

Among the ten RAF inhibitors used in this study, LY3009120, TAK-632, and PLX7904 have been reported as paradox-breaking inhibitors, which induce no or minimal paradoxical activation of the downstream MAPK pathway on the BRAF-wildtype background^12,16,17^. We examined the phosphorylation levels of MEK1/2 and ERK1/2 in BRAF-mutant, KRAS-mutant, or EGFR-mutant cancer cell lines after RAF inhibitor treatment to investigate the correlations between the dimerization potencies of the RAF inhibitors and their abilities to induce paradoxical activation of MEK-ERK signalling. The paradox-breaking inhibitors LY3009120, TAK-632, and PLX7904 were tested on these cell lines and compared with the effect of dabrafenib. In BRAF(V600E)-mutant COLO 205 cells, all four inhibitors showed concentration-dependent inhibition of MEK1/2 and ERK1/2 phosphorylation (Fig. 4a–d). In KRAS-mutant AsPC-1, A549, and EGFR-mutant H-1975 cells, paradoxical activation of MEK1/2 and ERK1/2 was substantially induced by dabrafenib (Fig. 4e,i,q), as previously reported^12,23^, and weakly induced by LY3009120 and TAK-632 (Fig. 4f,g,j,k,r,s). However, at higher concentrations, dabrafenib, LY3009120, and TAK-632 exerted inhibitory effects on MEK-ERK phosphorylation. In contrast, PLX7904 did not inhibit, but rather slightly activated MEK-ERK signalling at high concentrations in these KRAS- or EGFR-mutant cell lines (Fig. 4h,l,t). We also examined another KRAS-mutant cell line A427 and found that the effect on paradoxical activation was to a lesser degree (Fig. 4m–o). Moreover, we examined 293T cells overexpressing RAF isoforms and obtained the essentially same results of paradoxical activation as those observed in cancer cell lines (Supplementary Fig S4). These results are mostly but not completely consistent with previous studies^12,16,17^. In the study by Peng *et al*., LY3009120 did not show even a weak paradoxical activation in KRAS-mutant Calu6, HCT116, and NRAS-mutant SK-Mel30 cells^12^. In the study by Zhang *et al*., a high concentration (10 µM) of PLX7904 did not activate MEK-ERK signalling in A431 epidermoid carcinoma, SKBR3 breast cancer, and B9 cutaneous squamous cell carcinoma cell lines, all of which express wildtype BRAF^17^. The inconsistencies between the results from these previous studies and our findings may have resulted from the usage of different cell lines. Interestingly, inhibition of overexpressed CRAF, rather than BRAF or BRAF(V600E), paradoxically activated the MAPK pathway in 293T cells (Supplementary Fig. S4), indicating the major contribution of CRAF to paradoxical activation. Lastly, all these inhibitors showed no inhibitory effects on the downstream MAPK pathway (Supplementary Tables S4 and S5), indicating that the observed downstream inhibitory effects completely depend on RAF inhibition.

## Discussion

RAF dimerization is a key event in not only RAS-dependent RAF activation but also inhibitor-induced paradoxical activation of ERK signalling and even the development of drug resistance^3–8^. Co-immunoprecipitation is the method that is most widely used to detect RAF dimerization^3,5,7,16^, while ELISA^17^ and BRET^15^ have also been developed. In the present study, we utilized split luciferase complementation, a simple, rapid, and quantitative method compared to co-immunoprecipitation when used in the microplate format. In addition, bioluminescence assays, including split luciferase complementation, have many advantages over fluorescence assays such as FRET, BRET, and bimolecular fluorescence complementation; bioluminescence assays yield an extremely low background, a highly stable signal, and significantly higher sensitivity and are non-invasive in living cells, whereas fluorescence assays yield a high background and a strong but unstable signal and are invasive in cells due to the use of the external illumination, which causes phototoxic damage and bleaching^13^. Peng *et al*. have used split firefly luciferase complementation to detect RAF dimerization^12^. In contrast to their study, we used Emerald Luc (ELuc), an enhanced luciferase from the click beetle (*Pyrearinus termitilluminan*), which was shown in the previous studies to yields a significantly stronger and more stable light signal than firefly luciferase^11,13^. In addition, by screening probe pairs with different orientations of ELuc fragments and with various linker lengths, we obtained the optimal probe pairs that exhibited high sensitivity and a high signal-to-background ratio for the detection of BRAF homodimers, CRAF homodimers, and BRAF/CRAF heterodimers, including dimers with the BRAF(V600E) mutant (Fig. 1 and Table 1).

Based on the results of a screen of commercially available RAF inhibitors using our RAF dimer biosensor system, the system was successful in detecting dimerization. In 96-well microplate format, our assay system quantitatively detected inhibitor-induced RAF dimerization with high sensitivity (Fig. 2 and Supplementary Table S3), and the results were consistent with the previous reports^3,12,15–17^. It should be noted that the inhibitor doses which induced RAF dimerization in our assay system were around or lower than the reported clinical concentrations^24–26^, suggesting the assay system could be an useful tool to predict potential adverse effects in clinical uses. Moreover, the time course study performed using these biosensors revealed inhibitor-induced RAF dimerization with different temporal patterns depending on inhibitors and RAF isoform pairs (Fig. 3 and Supplementary Table S6). Dimerization induced within minutes indicates rapid membrane penetration of inhibitors and rapid conformational change of RAF proteins towards dimerization. In contrast, the subsequent slow and persistent increases in dimerization may result from slow membrane diffusion of inhibitors^27^ and/or an existence of a positive feedback loop from downstream MEK-ERK signalling^28,29^. Furthermore, the results that even single inhibitors, such as dabrafenib, vemurafenib, and TAK-632, induced different temporal patterns depending on RAF isoform pairs suggest that the feedback signalling may be inhibitor and RAF isoform-specific. Thus, the dimerization time course provides clues for understanding the mechanism of inhibitor-induced dimerization, and potentially, the correlation between dimerization and inhibition.

Dimerization and subsequent transactivation of RAFs are responsible for the paradoxical activation of ERK signalling by inhibitors^5–7^. Indeed, we reproduced the above finding by combining dimerization data obtained from 293T cells exogenously expressing RAF dimer-probes (Fig. 2) with the paradoxical activation data obtained from cancer cell lines harbouring EGFR, KRAS or BRAF mutation (Fig. 4) and 293T cells overexpressing RAF isoforms (Supplementary Fig. S4). On the BRAF-wildtype background, dabrafenib induced dimerization, resulting in paradoxical activation. In contrast, LY3009120 and TAK-632 induced medium to high levels of dimerization, but a subtle level of paradoxical activation, indicating these two as partial “paradox-breaking” inhibitors. Among the inhibitors tested in our study, PLX7904 is the only molecule that induced no RAF dimerization (Fig. 2) and no paradoxical activation of the downstream MAPK pathway on the BRAF-wildtype background (Fig. 4 and Supplementary Fig. S4), consistent to the original report by Zhang *et al*.^17^. They successfully dissociated paradoxical activation from inhibition of MAPK pathway by disrupting RAF dimerization. Thus, PLX7904 will be a good example showing that disrupting RAF dimerization is an effective strategy to overcome the paradox of RAF downstream activation.

Furthermore, our study showed that RAF inhibitors (dabrafenib, vemurafenib, sorafenib, regorafenib, LY3009120, SB-590885, MLN2480, TAK-632, and PLX4720) promoted dimerization with a common preference for CRAF-containing dimers (Fig. 2), strongly suggesting a major contribution of CRAF to paradoxical activation. Consistent with our observations, two groups have shown that paradoxical activation by PLX4720 and/or GDC-0879 depends on CRAF, but not BRAF, using knockdown or knockout studies^6,7^. Indeed, inhibition of overexpressed CRAF, rather than BRAF or BRAF(V600E), predominantly induced paradoxical activation in 293T cells (Supplementary Fig. S4). Taken together, CRAF, but not BRAF, likely plays a dominant role in the paradoxical activation of ERK signalling induced by not only PLX4720 and GDC-0879 but also most other RAF inhibitors, regardless of their different structural and biochemical properties.

In conclusion, our dimer detection system is simple, rapid, sensitive, and will be a powerful tool for drug screens to develop RAF inhibitors with minimal or no effects on dimerization, paradoxical activation, and resistance. Additionally, since our system can detect several combinations of RAF dimers [BRAF/BRAF, BRAF/CRAF, CRAF/CRAF, BRAF/BRAF(V600E), and CRAF/BRAF(V600E)], it will be a useful tool to study the mechanisms of drug-induced dimerization.

## Methods

### Compounds

Dabrafenib was purchased from Sequoia Research Products (Reading, UK). Vemurafenib, regorafenib, MLN2480 and SB-590885 were purchased from Selleckchem (Houston, TX, USA). PLX4720, PLX7904, and TAK-632 were purchased from MedKoo Biosciences Inc. (Morrisville, NC, USA). Sorafenib was synthesized by Carna Biosciences, Inc. (Kobe, Japan).

### Construction of mammalian expression vectors

A pcDNA3.1/myc-His vector encoding ELucN (Emerald Luc 1-415) and pcDNA4/V5-His vector encoding ELucC (Emerald Luc 394-542) with flexible GS linkers of various lengths are proprietary products of Carna Biosciences, Inc. and were originally constructed by Professor Ozawa and colleagues (The University of Tokyo, Japan). ELucN fused to BRAF or CRAF at the amino- or carboxy-terminus was obtained by subcloning the full-length human BRAF (NM_004333.3) or CRAF (NM_002880.3) cDNA into ELucN/pcDNA3.1/myc-His. Similarly, ELucC fused to BRAF or CRAF at the amino- or carboxy-terminus was obtained by subcloning the full-length human BRAF or CRAF cDNA into ELucC/pcDNA4/V5-His. The BRAF(V600E) mutant fused to ELucC was obtained by introducing a point mutation (1799t > a) into BRAF-ELucC using the QuikChange II Site-Directed Mutagenesis Kit (Agilent Technologies, Santa Clara, CA, USA).

### Cell culture

293T cells (ATCC CRL-3216) were cultured in high-glucose DMEM (Gibco 11960044) supplemented with 10% fetal bovine serum (FBS, GE Healthcare), GlutaMAX (Gibco), and 0.3% (v/v) penicillin-streptomycin (Gibco). COLO 205 (ATCC CCL-222), AsPC-1/CMV-Luc (JCRB #1454), and H-1975 (ATCC CRL-5908) cells were cultured in RPMI-1640, ATCC-modification (Gibco A10491-01), supplemented with 10% FBS and 0.3% (v/v) penicillin-streptomycin. A549 cells (ATCC CCL-185) were cultured in F-12K (ATCC 30-2004) supplemented with 10% FBS and 0.3% (v/v) penicillin-streptomycin. A427 cells (ATCC HTB-53) were cultured in Eagle’s MEM (ATCC) supplemented with 10% FBS and 0.3% (v/v) penicillin-streptomycin. Cells were maintained at 37 °C in a humidified atmosphere containing 5% CO_2_.

### RAF dimerization assay

The split luciferase complementation assay using Emerald Luc (ELuc), the enhanced luciferase from the click beetle (Brazilian *Pyrearinus termitilluminan*), was conducted using previously described methods^11^. 293T cells were seeded on 96-well white plates (Corning) one day prior to transfection and co-transfected with the pcDNA3.1/myc-His plasmid encoding ELucN-fused BRAF or CRAF and the pcDNA4/V5-His plasmid encoding ELucC-fused BRAF or CRAF using *Trans*IT-LT1 transfection reagent (Mirus Bio LLC, Madison, WI, USA). Approximately 24 hours after transfection, RAF inhibitors or vehicle control (DMSO) were added to the cells and incubated for 2 hours for concentration-dependence studies or for up to 24 hours for time course studies. The reaction was terminated by the removal of the culture medium and the immediate freezing of the entire culture plate. Luciferase activity was measured using an EnVision plate reader (Perkin Elmer) after adding 100 µl of NEO luciferase substrate reagent (TOYOBO) to each well and incubating the plate at room temperature for 30–40 minutes.

### MEK-ERK phosphorylation assay

Cells were treated with the indicated concentrations of RAF inhibitors for 2 hours and lysed in ice-cold RIPA buffer (Thermo Scientific) supplemented with EDTA-free Halt protease inhibitor (Thermo Scientific) and 1 mM EDTA (Invitrogen). After removing the cell debris by centrifugation, cell lysates were analysed by western blotting to assess the activation of MEK1/2 and ERK1/2 using phospho-MEK1/2 rabbit monoclonal antibody (clone 41G9, Cell Signaling Technology #9154, 1:1000 dilution), phospho-ERK1/2 rabbit monoclonal antibody (clone D13.14.4E, Cell Signaling Technology #4370, 1:2000 dilution), and HRP-conjugated goat anti-rabbit IgG (Invitrogen #G-21234, 1:2500 dilution). GAPDH was detected on the same blots using a GAPDH mouse monoclonal antibody (clone GA1R, Invitrogen #MA5-15738, 1:2000 dilution) and HRP-conjugated sheep anti-mouse IgG (GE Healthcare #NA931, 1:2500 dilution). The HRP signal was detected using Amersham ECL Select Western Blotting Detection Reagent (GE Healthcare). Images were captured by Amersham Imager 600 (GE Healthcare) and were analysed using ImageJ software (https://imagej.nih.gov/ij/). Membranes were cut out before blotting, and therefore full-length blots were not available. Uncropped images were shown in Supplementary Figs. S5 and S6.

### Equipment and settings

Luciferase activity was measured by EnVision plate reader (Perkin Elmer), using the ultrasensitive luminescence mode. Western blot images were acquired by Amersham Imager 600 (GE Healthcare) using the chemiluminescence mode with the autoexposure setting and analysed with ImageJ software (https://imagej.nih.gov/ij/).

### Statistical analysis

Data from RAF dimerization assays were analysed [means ± SD (standard deviation), n = 3] and plotted using Microsoft Excel software. Where indicated, data were analysed using one-way or two-way ANOVA with R statistical computing software (https://www.r-project.org/).

## Supplementary information

Supplementary Information

## Data Availability

All data reported in this paper are available from the corresponding author upon request.
