# Supplementary material for: Development of Highly Sensitive Biosensors of RAF Dimerization in Cells
Source: Sci Rep. 2019 Jan 24;9:636. doi: 10.1038/s41598-018-37213-2 (PMC6345758; doi:10.1038/s41598-018-37213-2)
Supplement: Supplementary file 1 — Supplementary Information [file 41598_2018_37213_MOESM1_ESM.pdf]

Supplementary Information for

"Development of Highly Sensitive Biosensors of RAF Dimerization in Cells"

Kyoko Miyamoto[1,*], Masaaki Sawa[1,2]

[1] CarnaBio USA, Inc., 329 Oyster Point Boulevard, Suite 300, South San Francisco, CA 94080, USA

[2] Carna Biosciences, Inc., 1-5-5 Minatojima-Minamimachi, BMA 3rd Floor, Chuo-ku, Kobe 650-0047, Japan

Supplementary Methods

Construction of split luciferase probes carrying BRAF(R509H) and CRAF(R401H) mutations

The BRAF(R509H) mutant fused to ELucC was obtained by introducing a point mutation (1526-1527ga>at) into BRAF-ELucC using the QuikChange II Site-Directed Mutagenesis Kit. CRAF(R401H) mutants fused to either ELucN or ELucC were obtained by introducing a point mutation (1202-1203gg>at) into CRAF-ELucN or CRAF-ELucC, respectively.

Cell viability assay

293T cells were seeded in a 96-black-well plate (Corning) one day before transfection and co-transfected with a pair of RAF split luciferase probe plasmids in the same manner as described in RAF dimerization assay. AlamarBlue (Bio-Rad) was added to the cell culture 24 or 48 hours after transfection, incubated for 3 hours, and the fluorescence was recorded at an excitation wavelength of 540 nm and an emission wavelength of 590 nm using an EnVision plate reader (Perkin Elmer). Results are presented as % differences compared to the cells co-transfected with empty vectors (pcDNA3.1/myc-His and pcDNA4/V5-His).

Kinase profiling

The inhibitory activities of RAF inhibitors towards BRAF, BRAF(V600E), CRAF(Y340D/Y341D), MEK1, MEK2, ERK1 and ERK2 were determined by performing a mobility shift assay (MSA) or ELISA with an ATP concentration near the Km or 1 mM using a commercial kinase profiling service performed by Carna Biosciences, Inc. (Kobe, Japan). Detailed information on the assay conditions is available on the Carna Biosciences website (https://www.carnabio.com/english/product/products-services.html).

MEK-ERK phosphorylation assay in 293T exogenous expression system

293T cells were single or co-transfected with pcDNA4/V5-His plasmid encoding BRAF or BRAF(V600E), and/or pcDNA3.1/myc-His plasmid encoding CRAF. Approximately 48 hours after transfection, cells were treated with the indicated concentrations of RAF inhibitors for 2 hours, and the lysates were collected and analysed in the same manner as described in the main text. Lysates from untreated cells were analysed to assess BRAF and CRAF expression levels using BRAF rabbit monoclonal antibody (clone D9T6S, Cell Signaling Technology #14814, 1:2000 dilution), CRAF mouse monoclonal antibody (clone D5X6R, Cell Signaling Technology #12552, 1:2000 dilution), V5-epitope tag mouse monoclonal antibody (Invitrogen #R960-25, 1:2000 dilution), and Myc-tag mouse monoclonal antibody (clone 9B11, Cell Signaling Technology #2276, 1:2000 dilution).

Supplementary Tables

Supplementary Table S1. Linker sequences of split luciferase probes used in Fig. 1a-d.

Name	Amino Acid Sequence
**ELucN-Linker6-BRAF**	GGGGDI
**ELucC-Linker6-BRAF**	GGGGDI
**ELucN-Linker6-CRAF**	GGGGGS
**ELucC-Linker6-CRAF**	GGGGDI
**BRAF-Linker7-ELucN**<br>**BRAF-Linker7-ELucC**<br>**CRAF-Linker7-ELucN**<br>**CRAF-Linker7-ELucC**	GSSGGGG

Supplementary Table S2. Linker sequences of split luciferase probes (BRAF-ELucN, BRAF-ELucC, CRAF-ELucN, and CRAF-ELucC) used in Fig. 1e-g.

Name	Amino Acid Sequence
**Linker 7**	GSSGGGG
**Linker 12**	GSSGGGGSGGGG
**Linker 17**	GSSGGGGSGGGGSGGGG
**Linker 22**	GSSGGGGSGGGGSGGGGSGGGG
**Linker 27**	GSSGGGGSGGGGSGGGGSGGGGSGGGG
**Linker 32**	GSSGGGGSGGGGSGGGGSGGGGSGGGGSGGGG

Supplementary Table S3. The results of one-way ANOVAs of the effects of inhibitor treatments on RAF dimerization.

			**BRAF/BRAF**		**BRAF/BRAF(V600E)**		**CRAF/BRAF**		**CRAF/BRAF(V600E)**		**CRAF/CRAF**	
**Treatment**	**df**	**Residuals**	***F***	***P***	***F***	***P***	***F***	***P***	***F***	***P***	***F***	***P***
**Dabrafenib**	3	8	59.86	**<0.001**	94.90	**<0.001**	232.6	**<0.001**	96.84	**<0.001**	562.9	**<0.001**
**Vemurafenib**	3	8	8.59	**<0.001**	43.23	**<0.001**	205.5	**<0.001**	31.28	**<0.001**	55.64	**<0.001**
**Sorafenib**	3	8	24.35	**<0.001**	579.4	**<0.001**	130.6	**<0.001**	211.4	**<0.001**	126.1	**<0.001**
**Regorafenib**	3	8	443.4	**<0.001**	799.5	**<0.001**	816.8	**<0.001**	273.4	**<0.001**	248.7	**<0.001**
**LY3009120**	3	8	1024	**<0.001**	172.9	**<0.001**	517.6	**<0.001**	508.8	**<0.001**	9447	**<0.001**
**SB-590885**	3	8	35.55	**<0.001**	35.55	**<0.001**	875.3	**<0.001**	491.8	**<0.001**	151.5	**<0.001**
**MLN2480**	3	8	94.94	**<0.001**	94.94	**<0.001**	519.3	**<0.001**	287.0	**<0.001**	30.58	**<0.001**
**TAK-632**	3	8	191.8	**<0.001**	191.8	**<0.001**	207.2	**<0.001**	233.5	**<0.001**	163.6	**<0.001**
**PLX4720**	3	8	6.30	**0.017**	3.87	0.056	38.69	**<0.001**	30.54	**<0.001**	10.15	**<0.001**
**PLX7904**	3	8	2.81	0.108	2.81	0.108	2.61	0.123	0.52	0.679	0.56	0.654

Data presented in Fig. 2 were analysed. df, degrees of freedom; *F*, *F* value; *P*, *P* value (n=3). Significant effects (P<0.05) are shown in bold.

Supplementary Table S4. IC_{50} values (μM) of RAF inhibitors against RAF isoforms and the MAPK pathways at near-Km concentrations of ATP (ATP_{low}).

	BRAF	BRAF (V600E)	CRAF (Y340D/Y341D)	MEK1	MEK2	ERK1	ERK2
**ATP (μM), Km/Bin**	0.061/0.1	3.2/5	0.39/0.5	11/10	13/15	34/50	33/50
**Dabrafenib**	0.0033	0.0036	0.0018	9.3595	>10	>10	>10
**Vemurafenib**	0.0523	0.4172	0.0895	>10	>10	>10	>10
**Sorafenib**	0.4157	0.4050	0.0610	>10	>10	>10	>10
**Regorafenib**	0.2945	0.2346	0.0678	>10	>10	>10	>10
**LY3009120**	0.0250	0.0129	0.0033	7.6610	>10	>10	>10
**SB-590885**	0.0007	0.0012	0.0011	>10	>10	>10	>10
**MLN2480**	0.4082	0.3405	0.0812	>10	>10	>10	>10
**TAK632**	0.0579	0.0643	0.0267	>10	>10	>10	>10
**PLX4720**	0.1058	1.0188	0.0129	2.6575	>10	>10	>10
**PLX7904**	0.0139	0.1753	0.1179	8.1603	>10	>10	>10

Assay platforms used in this experiment were MSAs for ERK1 and ERK2 and ELISAs for other kinases. Assays were performed in the presence of near-Km concentrations of ATP (indicated as Bin).

Supplementary Table S5. IC_{50} values (μM) of RAF inhibitors against RAF signalling cascades and the downstream MAPK pathways at 1 mM ATP (ATP_{high}).

	BRAF Cascade	BRAF(V600E) Cascade	CRAF (Y340D/Y341D) Cascade	MEK1 Cascade	MEK2 Cascade	ERK1	ERK2
**ATP (μM)**	1000	1000	1000	1000	1000	1000	1000
**Dabrafenib**	6.1839	0.2120	0.1562	>10	>10	>10	>10
**Vemurafenib**	>10	7.3320	1.3498	>10	>10	>10	>10
**Sorafenib**	>10	2.1137	1.1771	>10	>10	>10	>10
**Regorafenib**	>10	2.2891	1.3746	>10	>10	>10	>10
**LY3009120**	4.9167	0.2635	0.2735	>10	>10	>10	>10
**SB-590885**	0.2684	0.0052	0.1779	>10	>10	>10	>10
**MLN2480**	>10	4.6092	2.2608	>10	>10	>10	>10
**TAK-632**	>10	0.8707	3.4853	>10	>10	>10	>10
**PLX4720**	>10	>10	0.1102	>10	>10	>10	>10
**PLX7904**	3.9451	1.3154	2.2135	>10	>10	>10	>10

An MSA was performed to determine IC_{50} values in the presence of 1 mM ATP.

Supplementary Table S6. The results of two-way ANOVAs of the effects of the treatment type and time on RAF dimerization.

				Time		Treatment		Time:Treatment	
**Probe pair**	**Treatment**	**df**	**Residuals**	***F***	***P***	***F***	***P***	***F***	***P***
**BRAF/BRAF**	**Dabrafenib**	1	44	49.24	**<0.001**	228.61	**<0.001**	48.23	**<0.001**
	**Vemurafenib**	1	44	62.19	**<0.001**	141.47	**<0.001**	60.21	**<0.001**
	**LY3009120**	1	44	2.28	0.138	90.98	**<0.001**	2.11	0.153
	**PLX7904**	1	44	4.71	**0.036**	0.03	0.854	5.64	**0.022**
	**TAK-632**	1	44	6.82	**0.012**	118.31	**<0.001**	6.51	**0.014**
**BRAF/BRAF(V600E)**	**Dabrafenib**	1	44	46.12	**<0.001**	179.41	**<0.001**	41.26	**<0.001**
	**Vemurafenib**	1	44	76.27	**<0.001**	170.80	**<0.001**	64.56	**<0.001**
	**LY3009120**	1	42	10.59	**0.002**	106.39	**<0.001**	7.93	**0.007**
	**PLX7904**	1	44	58.08	**<0.001**	113.87	**<0.001**	35.71	**<0.001**
	**TAK-632**	1	44	4.30	**0.044**	106.84	**<0.001**	3.21	0.080
**CRAF/BRAF**	**Dabrafenib**	1	44	4.84	**0.033**	107.03	**<0.001**	6.46	**0.015**
	**Vemurafenib**	1	44	3.40	0.072	87.60	**<0.001**	9.47	**0.004**
	**LY3009120**	1	44	11.00	**0.002**	96.15	**<0.001**	13.27	**0.001**
	**PLX7904**	1	44	26.17	**<0.001**	0.81	0.372	0.46	0.501
	**TAK-632**	1	44	23.79	**<0.001**	89.97	**<0.001**	26.35	**<0.001**
**CRAF/BRAF(V600E)**	**Dabrafenib**	1	44	2.07	0.157	78.87	**<0.001**	3.23	0.079
	**Vemurafenib**	1	44	0.81	0.372	106.85	**<0.001**	8.75	**0.005**
	**LY3009120**	1	44	10.00	**0.003**	120.21	**<0.001**	11.87	**0.001**
	**PLX7904**	1	44	26.08	**<0.001**	0.86	0.358	1.19	0.281
	**TAK-632**	1	44	9.42	**0.004**	90.95	**<0.001**	11.33	**0.002**
**CRAF/CRAF**	**Dabrafenib**	1	47	9.55	**0.003**	80.05	**<0.001**	4.24	**0.045**
	**Vemurafenib**	1	47	35.15	**<0.001**	40.73	**<0.001**	11.12	**0.002**
	**LY3009120**	1	47	31.10	**<0.001**	99.73	**<0.001**	16.05	**<0.001**
	**PLX7904**	1	47	6.41	**0.015**	0.23	0.631	1.29	0.263
	**TAK-632**	1	47	108.92	**<0.001**	96.59	**<0.001**	67.30	**<0.001**

Data presented in Fig. 3 were analysed. df, degrees of freedom; *F*, *F* value; *P*, *P* value (n=3). Significant effects ($P<0.05$) are shown in bold.

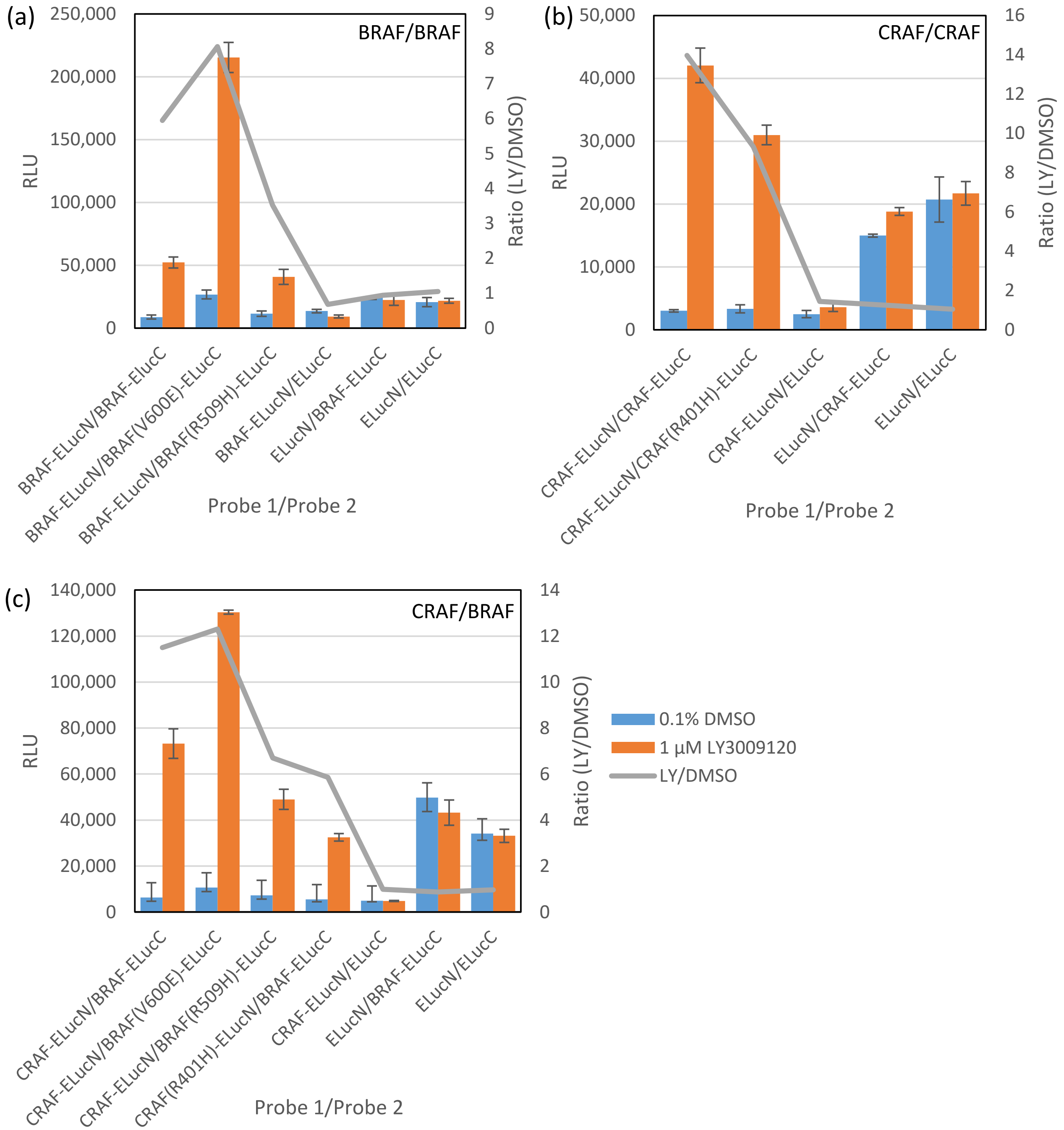

Supplementary Fig. S1. RAF split luciferase assay paired with dimerization-impaired mutants or unfused ELuc fragments.

293T cells were transiently co-transfected with the indicated pairs of plasmids and treated with 1 µM LY3009120 or 0.1% DMSO for 2 hours. Luciferase activities were measured, and the results are presented as RLUs (means±SD, n=3) and the ratio of RLUs measured in LY3009120-treated cells to DMSO-treated cells. Pairing with dimerization-impaired mutant, BRAF(R509H) or CRAF(R401H), resulted in a reduced LY/DMSO ratio compared to wildtype RAF proteins. The LY3009120 treatment did not increase luciferase activities in cells paired with unfused ELucN or ELucC compared to the DMSO control.

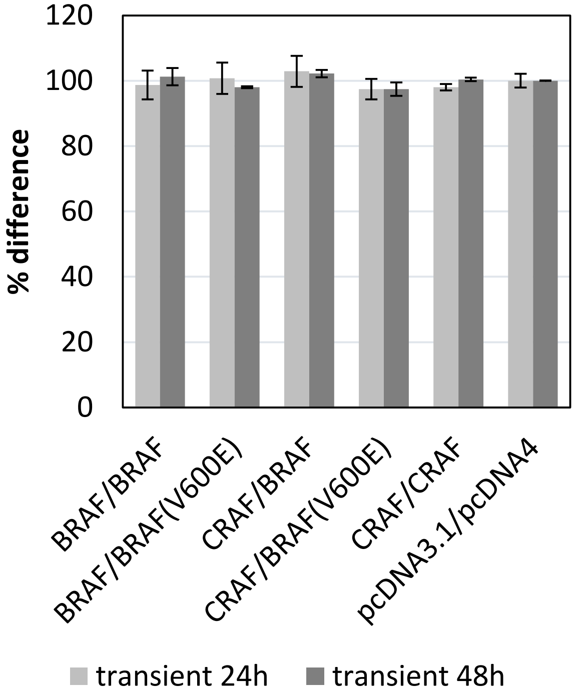

Supplementary Fig. S2. Viability of 293T cells transiently transfected with RAF split luciferase probes. The viability of 293T cells transiently transfected with RAF split luciferase probes was analysed using AlamarBlue staining. Data are presented as % differences compared to vector-co-transfected cells (means±SD, n=2).

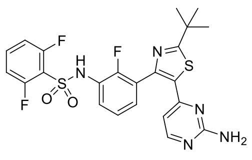

Dabrafenib

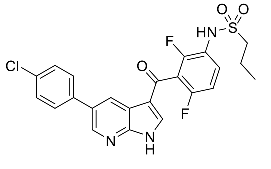

Vemurafenib

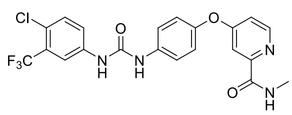

Sorafenib

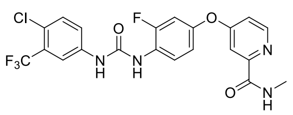

Regorafenib

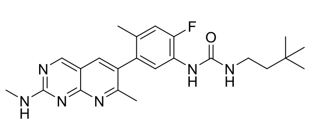

LY3009120

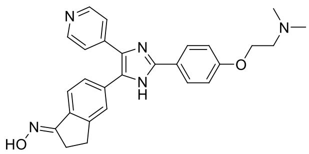

SB-590885

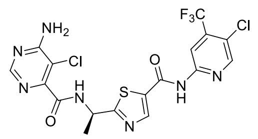

MLN2480

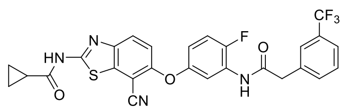

TAK-632

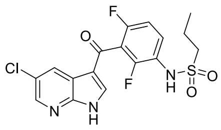

PLX4720

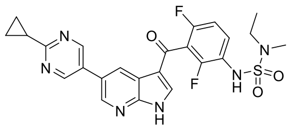

PLX7904

Supplementary Fig. S3. Chemical structures of RAF inhibitors used in this study.

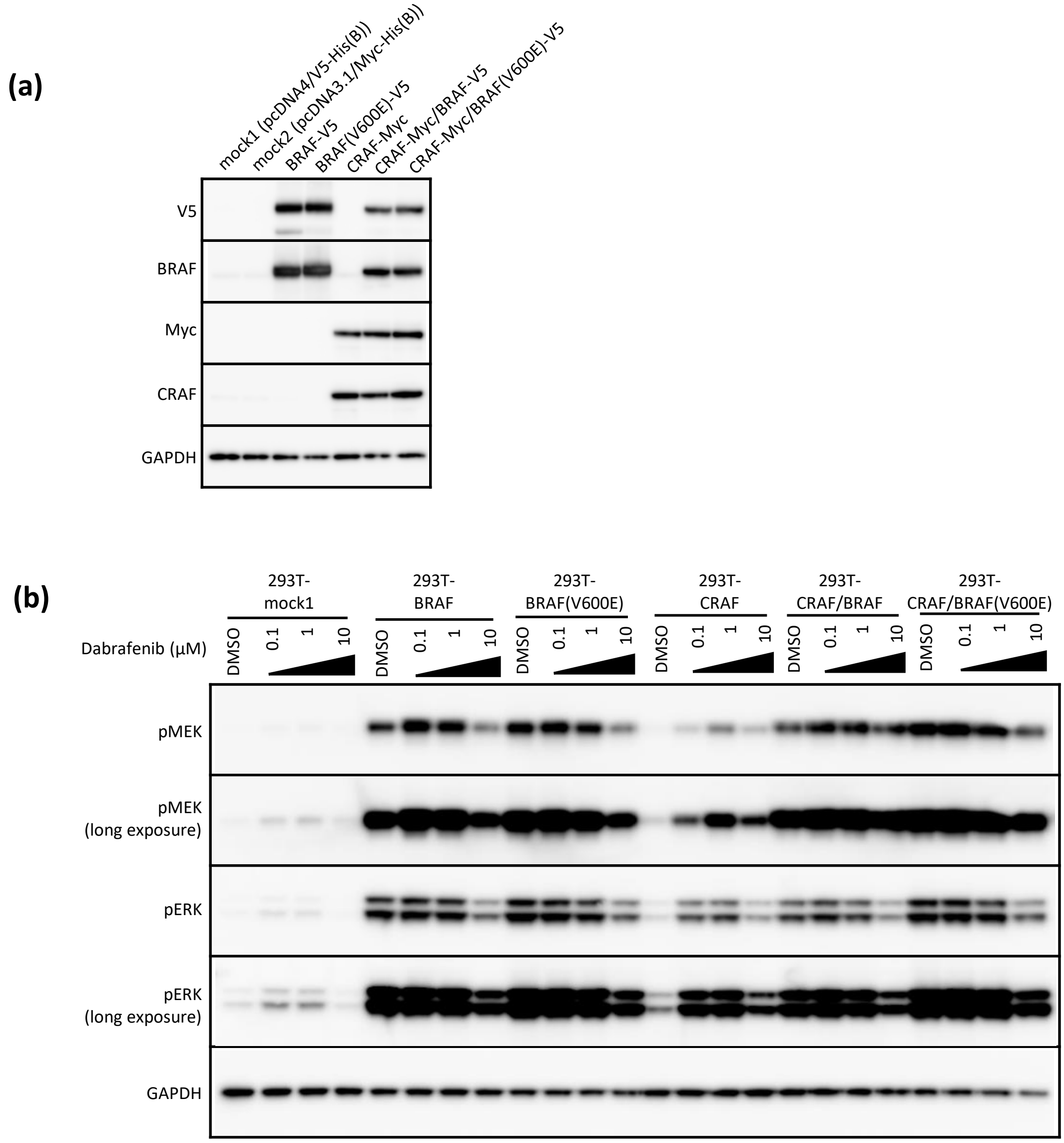

Supplementary Fig. S4. Effects of RAF inhibitors on the MAPK pathway in 293T cells transiently overexpressing BRAF, BRAF(V600E), and/or CRAF. (a) BRAF and CRAF expression levels in 293T cells 48 hours after transfection of BRAF, BRAF(V600E), and/or CRAF-encoding plasmids. **(b)** Phospho-MEK1/2, phospho-ERK1/2, and GAPDH expression levels in BRAF, BRAF(V600E), and/or CRAF-overexpressing 293T cells with a 2 hour-treatment of dabrafenib. **(c)** Phospho-MEK1/2, phospho-ERK1/2, and GAPDH expression levels in BRAF, BRAF(V600E), and/or CRAF-overexpressing 293T cells with a 2 hour-treatment of the indicated inhibitors. Solid black lines represent the boundaries of images cropped from different blots. The membranes were cut out before blotting, and full-length blots are unavailable. Uncropped images corresponding to (a) and (b) are shown in Supplementary Fig. S6.

Continued from Supplementary Fig. S4

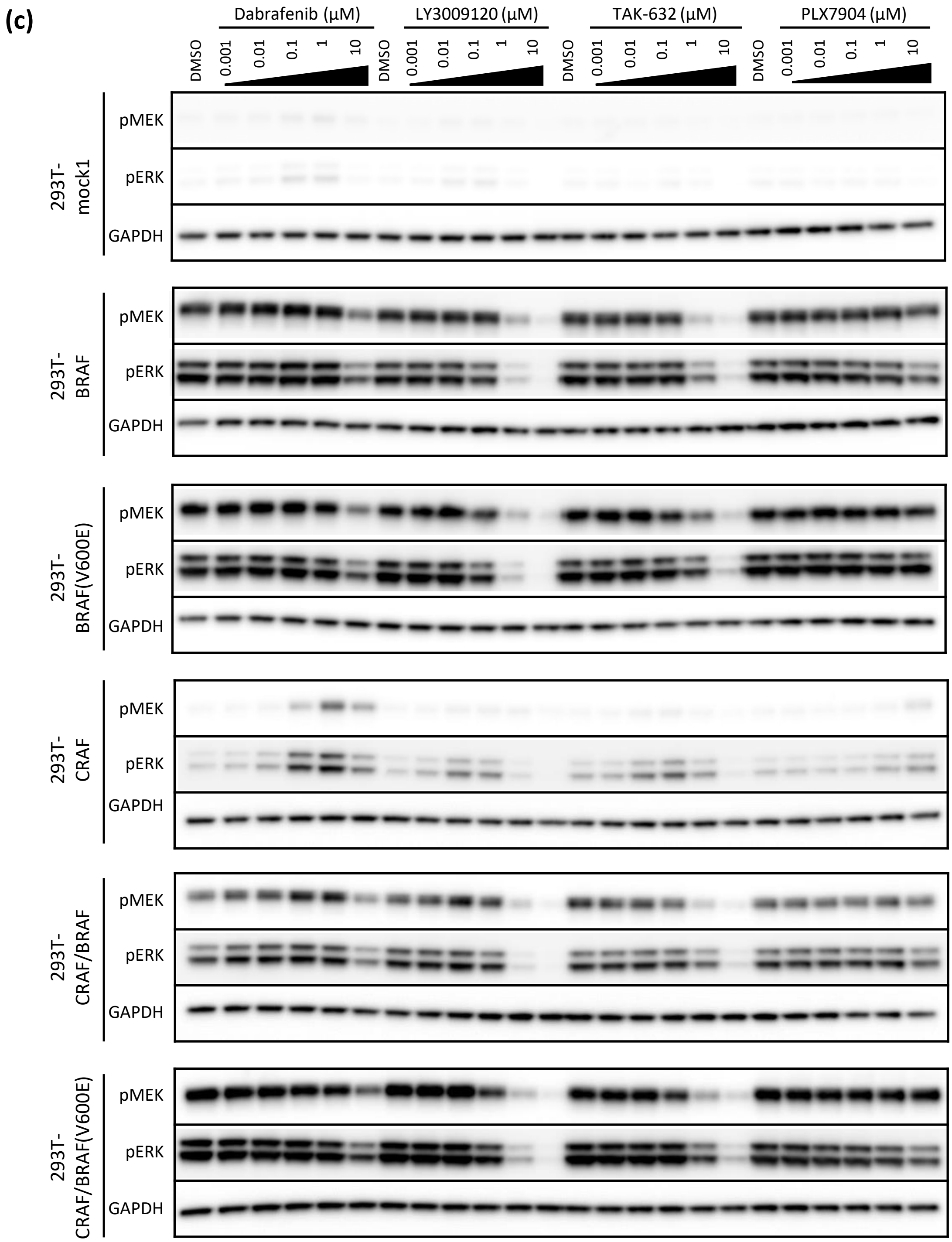

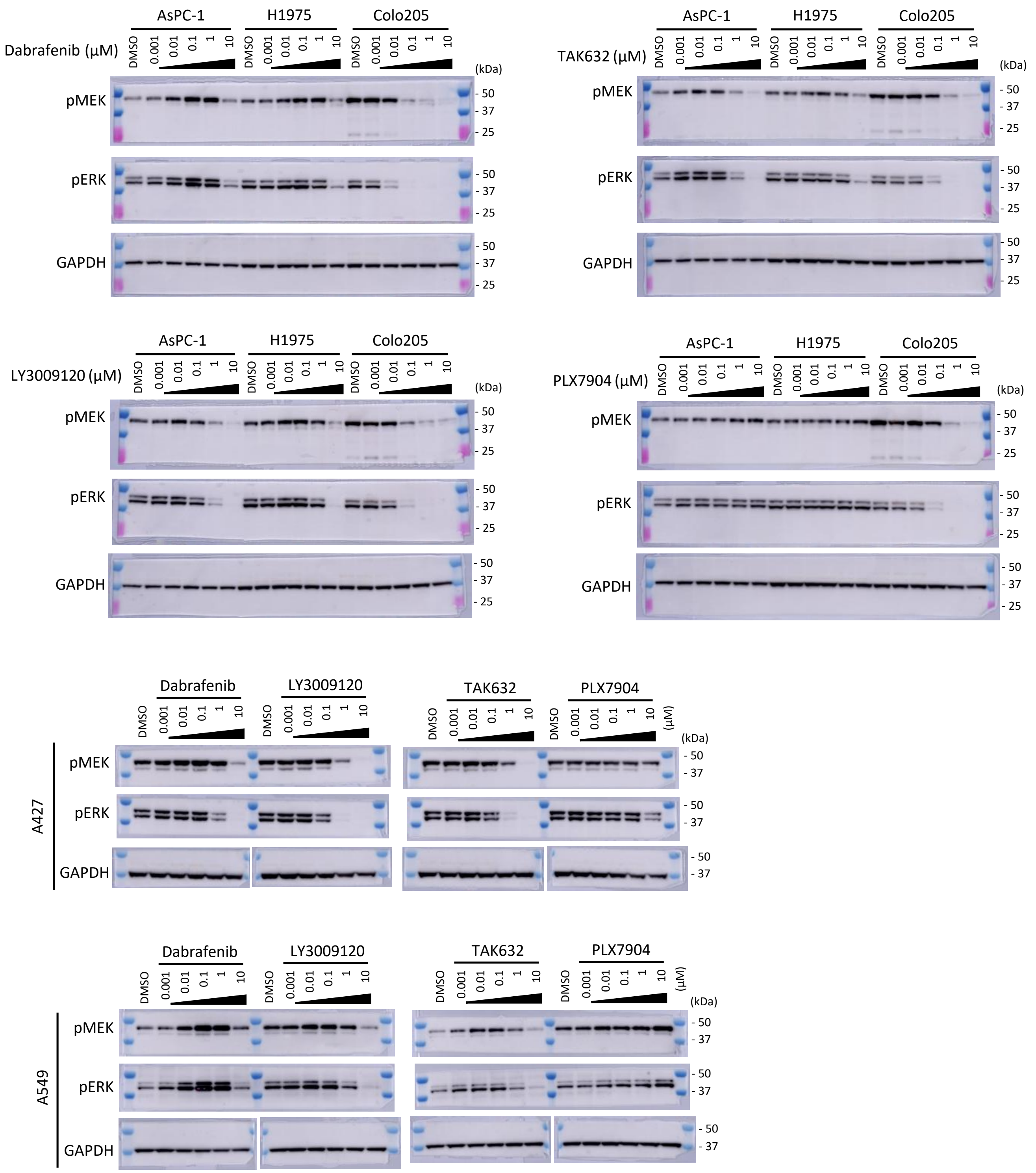

Supplementary Fig. S5. Uncropped western blot images corresponding to Fig. 4. Blot images merged with colorimetric images are shown. Merged images were acquired right after the acquisition of blot images used in Fig. 4.

(a)

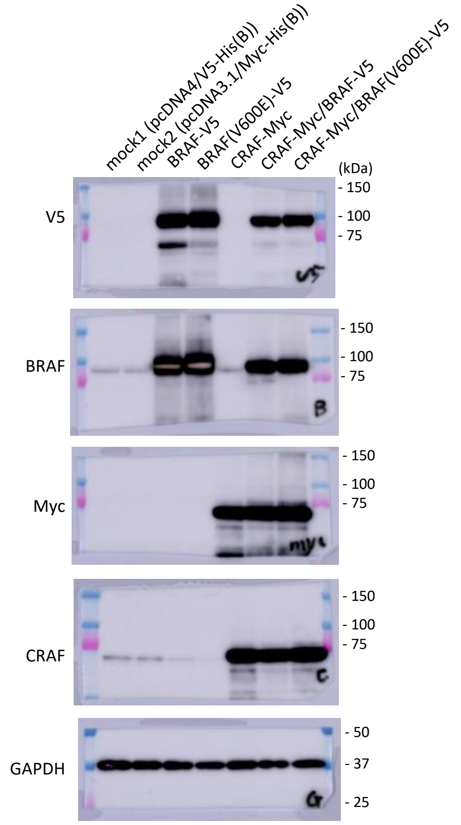

(b)

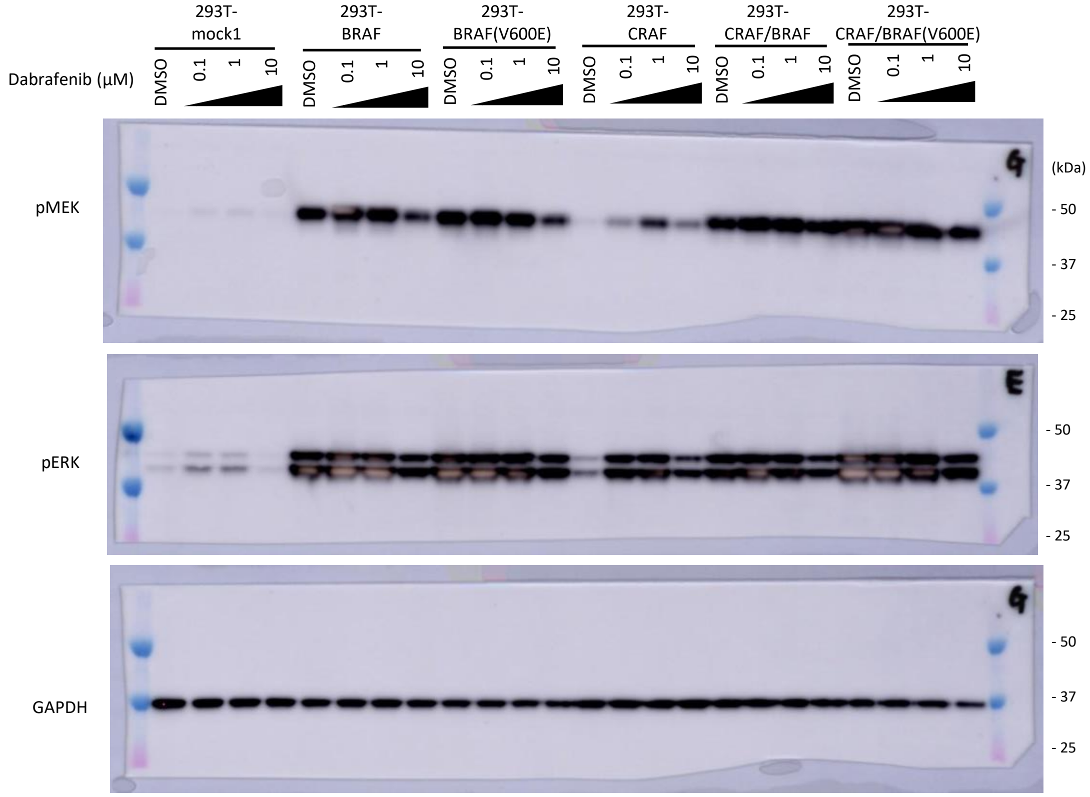

Supplementary Fig. S6. Uncropped western blot images corresponding to Supplementary Fig. S4a-b. Blot images merged with colorimetric images are shown. Merged images were acquired right after the acquisition of blot images used in Supplementary Fig. S4a-b.
